# Potential for Radiation Dose Reduction in Dual-Source Computed Tomography of the Lung in the Pediatric and Adolescent Population Compared to Digital Radiography

**DOI:** 10.3390/diagnostics11020270

**Published:** 2021-02-10

**Authors:** Matthias Wetzl, Matthias Stefan May, Daniel Weinmann, Matthias Hammon, Markus Kopp, Renate Ruppel, Regina Trollmann, Joachim Woelfle, Michael Uder, Oliver Rompel

**Affiliations:** 1 Department of Radiology, University Hospital Erlangen, 91054 Erlangen, Germany; matthias.may@uk-erlangen.de (M.S.M.); daniel_weinmann90@web.de (D.W.); matthias.hammon@gmail.com (M.H.); markus.kopp@uk-erlangen.de (M.K.); michael.uder@uk-erlangen.de (M.U.); oliver.rompel@uk-erlangen.de (O.R.); 2 Imaging Science Institute, University Hospital Erlangen, 91054 Erlangen, Germany; 3 Department of Pediatrics and Adolescent Medicine, University Hospital Erlangen, 91054 Erlangen, Germany; renate.ruppel@uk-erlangen.de (R.R.); regina.trollmann@uk-erlangen.de (R.T.); joachim.woelfle@uk-erlangen.de (J.W.)

**Keywords:** DSCT, tin filtration, low-dose, lung, pediatric

## Abstract

Low-dose dual-source computed tomography (DSCT) protocols for the evaluation of lung diseases in children and adolescents are of importance since this age group is particularly prone to radiation damage. The aim of this study was to evaluate image quality of low-dose DSCT of the lung and to assess the potential of radiation dose reduction compared to digital radiographs (DR). Three groups, each consisting of 19 patients, were examined with different DSCT protocols using tin prefiltration (Sn96/64/32 ref. mAs at 100 kV). Different strengths of iterative reconstruction were applied (ADMIRE 2/3/4). DSCT groups were compared to 19 matched patients examined with posterior–anterior DR. Diagnostic confidence, detectability of anatomical structures and small lung lesions were evaluated on a 4-point Likert scale (LS 1 = unacceptable, 4 = fully acceptable; a value ≥ 3 was considered acceptable). Effective dose (ED) was 31-/21-/9-fold higher in Sn96/Sn64/Sn32 compared to DR. Diagnostic confidence was sufficient in Sn96/Sn64 (LS 3.4/3.2), reduced in Sn32 (LS 2.7) and the worst in DR (LS 2.4). In DSCT, detectability of small anatomical structures was always superior to DR (*p* < 0.05). Mean lesion size ranged from 5.1–7 mm; detectability was acceptable in all DSCT groups (LS 3.0–3.4) and superior to DR (LS 1.9; *p* < 0.05). Substantial dose lowering in DSCT of the pediatric lung enables acceptable detectability of small lung lesions with a radiation dose being about 10-fold higher compared to DR.

## 1. Introduction

Dual-source computed tomography (DSCT) for the evaluation of lung diseases in children and adolescents is well established. Because of the high radiation sensitivity of the pediatric population with the potential risk of radiation-induced damage, careful handling is mandatory [1,2]. According to the ALARA principle (“as low as reasonably achievable”), radiation dose should be reduced as much as possible without impairing the diagnostic value. However, a decrease in image quality in concomitance with lower radiation doses might be tolerable.

There are different approaches to decreasing radiation exposure in computed tomography. One of the most effective methods is the reduction of tube voltage because the dose increases with the square of the tube voltage. On the other hand, it varies approximately linearly with tube current [3]. Tin prefiltration is another technique to reduce radiation dose.

Lately, third-generation DSCT scanners are equipped with additional tin prefiltration that removes low energy photons of the X-ray beam. These photons contribute little to the image quality but increase the radiation burden. The so-called “spectral shaping” has the potential to reduce radiation dose in several anatomical regions in adult and pediatric patients [4,5,6,7].

Advanced iterative reconstruction algorithms can preserve image quality in pediatric computed tomography (CT) examinations with low-dose examination protocols [8]. Newell et al. reported a phantom study indicating that third-generation DSCT scanners using iterative reconstruction methods (ADMIRE, Siemens Healthcare, Erlangen, Germany) can generate accurate quantitative CT images with acceptable image noise at very low dose levels [9]. In a study by Rompel et al., chest CT angiography in newborns and young children performed with a third-generation DSCT scanner using a 70-kV protocol, together with stronger reconstruction levels of ADMIRE, allowed high image quality at a low radiation dose level [10].

The aim of this retrospective, comparative study was to assess image quality, lesion detectability and the potential of radiation dose reduction in pediatric lung DSCT using spectral shaping and ADMIRE compared to DR.

## 2. Materials and Methods

This study was conducted in accordance with the guidelines of the Declaration of Helsinki and approved by our local ethics committee (ethics committee of Friedrich-Alexander Universität Erlangen/Nürnberg, protocol code 36_14 B, date of approval 12 March 2014). Written informed consent for DSCT of the chest was obtained for all patients. The institutional review board waived supplemental agreement because of the retrospective study design.

### 2.1. Patient Characteristics

A total of 57 patients with DSCT examinations of the lung were enrolled in this study. They were retrospectively selected from 3 examination protocols with tin prefiltration (Sn96/64/32). Each study group consisted of 19 patients (Table 1). For comparison of dose and image quality, 19 patients examined with posterior–anterior DR were selected from our institutional database. The patients had been referred for CT or DR to further investigate suspected or known noncancer lung diseases such as cystic fibrosis, primary ciliary dyskinesia, prolonged course of pneumonia, chronic lung complications of pneumonia, suspected pulmonary hemorrhage, aspiration pneumonitis, pulmonary Langerhans cell histiocytosis, tuberculosis and atelectasis or pleural effusion of unclear origin. All groups were matched with respect to age, weight and body mass index. As shown in Table 1, there were no significant differences in patient characteristics.

### 2.2. DSCT with Tin Prefiltration

All low-dose DSCT examinations were performed using a third-generation scanner (Somatom Definition Force, Siemens Healthcare, Erlangen, Germany) with 0.6 mm tin prefiltration. CT parameters were as follows: 0.25 s gantry rotation time, detector collimation of 2 × 96 × 0.6 mm, slice collimation of 192 × 0.6 mm using z-flying focal spot technique, spiral pitch factor 3.0 and tube voltage modulation switched off. Since tin prefiltration is only available at 100 and 150 kV tube voltage, with higher diagnostic dose efficiency at 100 kV [11], the lower kV setting was used for this study. Automatic exposure control (CareDose 4D, Siemens Healthcare, Erlangen, Germany) was used with a reference tube current time product per rotation of 96, 64 and 32 mAs. Dose requirement of these examination protocols was 17, 11 and 6% of an in-house full-dose protocol without tin prefiltration (100 kV, 64 mAs), reinforcing the ability of tin prefiltration to approximately achieve a 90% reduction of radiation dose [5,12]. Examinations were performed in supine position with elevated arms from the upper to the lower thoracic aperture. If necessary, a bodyweight-adapted dose of iodinated contrast medium was injected intravenously.

### 2.3. DSCT Postprocessing

Images of all DSCT groups were reconstructed with a slice thickness of 1.0 mm using filtered back-projection (FBP) as well as advanced model iterative reconstruction with a medium, an intermediate and a strong increment (ADMIRE strengths 2/3/4). For all images, a dedicated lung convolution kernel (Bl57) was used, as recommended by the manufacturer. Iterative reconstruction is characterized by repeated forward and back projection of raw data and image data in combination with statistical modeling. The repeated comparison of projected raw data with the measured data allows removal of geometric imperfections. ADMIRE is built upon these principles, with substantial modifications, allowing a high iteration speed [9]. It has previously been shown that ADMIRE has the potential to significantly improve image quality while reducing noise and artifacts in CT scans [10,13,14]. In ADMIRE, images are reconstructed by minimizing the objective function incorporated with an accurate system model, a statistical noise model and a prior model [15].

### 2.4. Posterior–Anterior DR

Nineteen DR of the chest in a single posterior–anterior projection at full inspiration were included in the study. The examinations were acquired with a dedicated needle detector system (DX-S, Agfa, Mortsel, Belgium), equipped with the Multiscale Image Contrast Amplification (MUSICA). Tube voltage setting was 80 kV in children 4–8 years old and 120 kV in older children and adolescents. Dose-reducing aluminum (1 mm) and copper (0.1 mm) filter systems were deployed in younger children. Automatic exposure control as well as a scattered radiation grid were applied in patients 8 years and older.

### 2.5. Image Analysis of DSCT and DR

All images were anonymized and transferred to a postprocessing 3D console (SyngoVia VA30A, Siemens Healthcare, Erlangen, Germany). Image analysis was performed on the postprocessing 3D console in randomized order independently by two radiologists (O.R. and M.H., with 25 and 10 years of experience in pediatric lung CT and radiography).

DSCT images were interpreted in axial, coronal and sagittal orientation with 1 mm slice thickness. Multiplanar reconstructions and maximum and minimum intensity projections were allowed to be used at the discretion of the readers. The default window setting was center −600 HU and width 1700 HU and could be individually adjusted by the readers. Window settings of RGs could be adjusted too.

Image quality of the DSCT reconstructions (FBP, ADMIRE 2,3,4) and DR was interpreted subjectively, following the European Guidelines on Quality Criteria.

Overall diagnostic confidence as well as detectability of the following anatomical structures were classified on a 4-point Likert scale (LS 1 = unacceptable, 2 = acceptable under limited conditions, 3 = probably acceptable and 4 = fully acceptable): medium-sized and small pulmonary vessels, lung fissures, lung parenchyma and tertiary bronchi.

Suspicious lung lesions were subjectively rated with respect to detectability, contrast and contour sharpness, using the above-mentioned 4-point Likert scale.

Concerning detectability of anatomical structures and suspicious lesions, a cut-off value of 3 points on the 4-point Likert scales was defined for acceptance in our study. This was also true for overall diagnostic confidence.

### 2.6. DSCT Radiation Dose

Radiation dose exposure was assessed as volumetric CT dose index (CTDI_vol_) and dose length product (DLP). Estimated effective dose (ED) was calculated as

ED = DLP ∗ k_i,_ (1)

using an individual linear interpolation of the conversion factor reported in literature for chest CT at 100 kV between neonates (k_0_ = 0.0739 mSv/mGy∗cm), 1-year-olds (k_1_ = 0.048 mSv/mGy∗cm), 5-year-olds (k_5_ = 0.0322 mSv/mGy∗cm), 10-year-olds (k_10_ = 0.0235 mSv/mGy∗cm) and 18-year-olds/adults (k_adult_ = 0.0144 mSv/mGy∗cm) as a function of days of life [10,16].

### 2.7. DR Radiation Dose

Radiation dose exposure was assessed as entrance dose (D_E_), which can be calculated as the ratio of dose area product (DAP) and field size (A). For estimation of effective dose (ED) from D_E_, Seidenbusch et al. provide conversion factors as a function of age and tube voltage (k_AV_) for pediatric chest radiographs [17]. Conversion factors (k_AV_ in mSv/mGy) were 0.46 for neonates (80 kV), 0.43 for 1-year-old infants (80 kV), 0.38 for 5-year-old children (80 kV), 0.45 for 10-year-old children (120 kV) and 0.42 for 15-year-old patients and adults (120 kV). ED was calculated as

ED = D_E_ ∗ k_AV_. (2)

### 2.8. Statistical Analysis

Statistical analysis was performed using SPSS software version 25 (IBM, Armonk, New York, NY, USA). Values are given as mean ± standard deviation if normal distribution was assumed by Kolmogorow–Smirnov tests. Otherwise, median and range values were added. Categorical variables are expressed as frequencies and percentages. For multiple comparison, ANOVA with Bonferroni and Games-Howell tests was applied. Equality of variance was determined by Levene’s test. All *t*-tests were performed 2-sided, and *p* < 0.05 was considered to be statistically significant. Inter-rater agreement was assessed using Cohen’s kappa test. Values > 0.61 were interpreted as substantial and >0.81 as almost in perfect agreement according to Landis and Koch [18].

## 3. Results

### 3.1. Diagnostic Confidence

Overall diagnostic confidence was best with ADMIRE 4 in all DSCT groups and iterative reconstruction was always superior to FBP. Corresponding values are given in Table 2. With the use of ADMIRE 3/4 in the Sn96 group (3.3 ± 0.5/3.4 ± 0.5) and ADMIRE 4 in the Sn64 group (3.2 ± 0.4), acceptable overall diagnostic confidence was achieved (LS ≥ 3). In the Sn32 group (LS = 2.7 ± 0.5 for ADMIRE 4) and with DR (LS = 2.4 ± 0.5), the defined cut-off value of 3 points on the 4-point Likert scale was not attained (Sn96_ADM4_ vs. Sn32_ADM4_: *p* < 0.001; Sn64_ADM4_ vs. Sn32_ADM4_: *p* = 0.013; DR vs. Sn96 _ADM4_/Sn64_ADM4_: *p* < 0.001; DR vs. Sn32_ADM4_: *p* = 0.505).

### 3.2. Anatomical Structures

**Figure 1:**
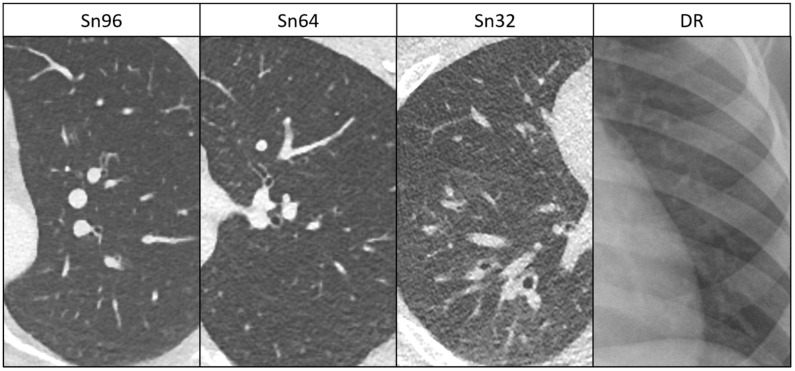
Delineation of anatomical structures in low-dose DSCT (ADMIRE 4) and DR. Sn96: 18-year-old boy, Sn64: 17-year-old girl, Sn32: 14-year-old boy and DR: 11-year-old boy. Delineation of anatomical structures such as bronchial walls is acceptable in the Sn96 and Sn64 groups and partially limited in the Sn32 group, but it is always superior compared to DR.

Detectability of anatomical structures improved with increasing strength levels of ADMIRE. Detectability also differed between the DSCT groups. When using ADMIRE 4, it was always superior to DR (Table 2). A pictorial example is given in Figure 1. When image reconstruction was performed with ADMIRE 4, corresponding values were 3.2 ± 0.6/3.1 ± 0.4/2.5 ± 0.5/1.9 ± 0.6 in the Sn96/Sn64/Sn32/DR group for small vessels (DR vs. Sn96/Sn64: *p* < 0.001; DR vs. Sn32: *p* = 0.004), 3.8 ± 0.4/3.7 ± 0.4/3.5 ± 0.5/1.6 ± 0.4 in the Sn96/Sn64/Sn32/DR group for tertiary bronchi (DR vs. Sn96/Sn64/Sn32: *p* < 0.001) and 2.9 ± 0.5/2.9 ± 0.4/2.7 ± 0.5/1.2 ± 0.3 in the Sn96/Sn64/Sn32/DR group for lung parenchyma (DR vs. Sn96/Sn64/Sn32: *p* < 0.001). With ADMIRE 4, acceptable detectability of small vessels was achieved in the Sn96 and Sn64 groups. This was also true for tertiary bronchi in all groups. For lung parenchyma, the defined cut-off value of 3 points was not attained in any group.

### 3.3. Suspicious Lung Lesions

In the Sn96/Sn64/Sn32/DR group, 74/68/67/68 suspicious lung lesions were found. Mean lesion sizes were in the range of 5.1–7.0 mm without significant differences between the groups (Table 3). The lesions comprised subpleural, peribronchovascular or centrilobular nodules, mucoid impaction, tree-in-bud opacities, septal thickening, local ground-glass opacity, circumscribed consolidations, abscess formation, bronchiectasis, pneumatoceles and cavitations in DSCT and bronchiectasis, septal thickening, mucoid impactions, nodules and consolidations in DR.

**Figure 2:**
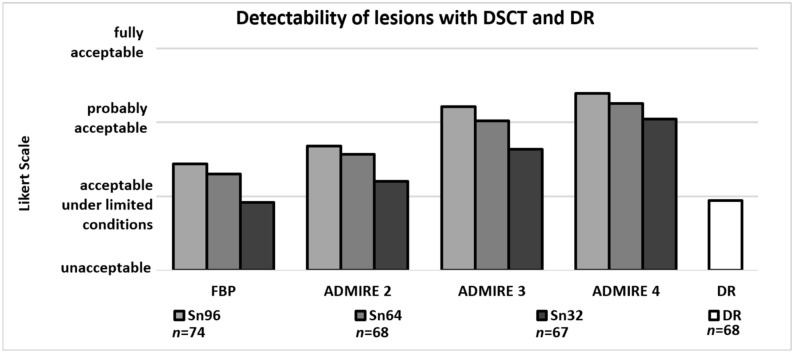
Detectability of suspicious lesions (*n*) with DSCT dependent on dose groups and iterative reconstruction set and DR. Detectability of lesions increases with rising levels of ADMIRE. With ADMIRE 4, detectability with DSCT was always acceptable (score of 3 or higher) and was significantly superior compared to DR.

**Figure 3:**
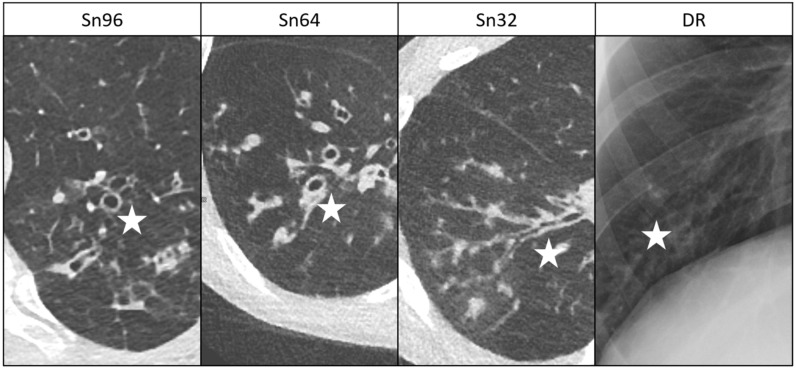
Comparative detectability of lung lesions in patients with cystic fibrosis in low-dose DSCT (ADMIRE 4) and DR. **Sn96:** 13-year-old girl, **Sn64:** 10-year-old boy, **Sn32:** 17-year-old boy and **DR:** 17-year-old boy. Bronchiectasis, bronchial wall thickening and mucoid impactions are marked with an asterisk. Detectability of lung lesions is acceptable in all DSCT groups and limited in DR.

Detectability, contrast and contour sharpness of suspicious lung lesions decreased in the DSCT groups with decreasing current time products but were always superior to DR. For ADMIRE 4, detectability of lesions was rated 3.4 ± 0.6/3.3 ± 0.7/3.0 ± 0.6 in the Sn96/Sn64/Sn32 group, compared to 1.9 ± 0.8 for DR (DR vs. Sn96/Sn64/Sn32: *p* < 0.001; Figure 2). Thus, with the use of ADMIRE 4, acceptable detectability of small lesions was achieved in all DSCT groups. For corresponding values of significance see Table 3. An example is given in Figure 3. As for all ratings, inter-rater agreement was almost perfect (k value > 0.81).

### 3.4. Radiation Dose

Radiation dose measurements are summarized in Table 4. Mean effective dose (ED) was 0.219 ± 0.096/ 0.149 ± 0.054/ 0.065 ± 0.035 mSv in the Sn96/ Sn64/ Sn32group (Sn96 vs. Sn64 vs. Sn32: *p* < 0.05).

For DR in the posterior–anterior projection, mean ED was 0.007 ± 0.003 mSv.

Therefore, mean ED was about 31-/ 21-/ 9-fold higher in the Sn96/ Sn64/ Sn32 DSCT group compared to DR in a single plane.

## 4. Discussion

This retrospective study assessed the potential of radiation dose reduction in DSCT of the pediatric and adolescent lung using spectral shaping together with iterative reconstruction compared to DR.

In the Sn96 and Sn64 DSCT groups, an acceptable diagnostic confidence and detectability of suspicious lung lesions were found (LS ≥ 3), and they were significantly superior to DR. The two aforementioned DSCT protocols are known to require about 17%/11% of the dose of an in-house standard CT protocol without spectral shaping [12]. In our present study, radiation doses of the Sn96 and Sn64 groups turned out to be 31- and 21-fold higher compared to DR.

In the Sn32 group, an acceptable Likert scale level for diagnostic confidence was barely missed and detectability of small anatomical structures was partially limited. This should be due to increased noise levels. Nevertheless, detectability of suspicious lung lesions was acceptable when a strong increment of iterative reconstruction (ADMIRE 4) was used. This underlines that iterative reconstructions seem to be essential at the lowest CT groups. Moreover, Sn32 significantly outperformed DR concerning lesion detectability, contrast and contour sharpness. The Sn32 protocol is known to require about 6% of the dose of our standard CT protocol mentioned before and turned out to be 9-fold higher compared to DR.

We found no other study that compared low-dose DSCT examinations using spectral shaping and ADMIRE with DR in the age group of children and young adolescents. The main strength of this study consists in detailed identification of dose requirements of different low-dose DSCT protocols compared to DR with the latter serving as a benchmark.

Direct comparability of diagnostic confidence between CT and DR examinations needs to be looked at critically, and radiologists would usually favor any quality of CT imaging towards plain radiographs. However, in our study, we were the first to clearly prove superiority of low-dose DSCT over DR with respect to diagnostic confidence as well as detectability of anatomical and pathological structures.

Chest CT examinations in adults with radiation exposure close to X-ray examinations proved their potential in a study by Kroft et al. [19]. Mean perceived confidence for diagnosis was 88% for radiographs and 98% for ultra-low-dose CT. Furthermore, ultra-low-dose CT added value for diagnosis in 40 of 200 patients. Radiation dose was 0.040 mSv in 2-plain radiographs compared to 0.071 mSv in CT. Ebner et al. investigated chest phantoms with artificial lung nodules between 5 and 12 mm at a mean dose level of 0.13 mSv. In their analysis, sensitivity was 96.2% for micro-dose CT and 75% for radiographs. They conclude that micro-dose CT has the potential to replace conventional chest radiography for lung nodule detection [20].

Miéville et al. examined the effect of iterative reconstructions on image quality in low-dose CTs in children with cystic fibrosis [21]. In accordance with our study, they found out that small structures are better visible in low-dose CT examinations when iterative reconstructions are applied. In a study by Weis et al. [22], a 100 kV pediatric chest CT protocol using spectral shaping (Sn100 kV) was compared with a 70 kV standard protocol. Significant dose reduction up to 0.21 mSv and superior subjective image quality of lung structures were achieved with the Sn100 kV protocol. Consequently, their dose results resemble the mean radiation doses of the Sn96 group in our study. In a phantom study, Martini et al. [23] analyzed solid and subsolid lung lesions with low-dose protocols, resulting in effective doses that were comparable to ours (0.14 mSv at 1/8th and 0.05 mSv at 1/20th of standard dose). They reached diagnostic image quality when using ADMIRE 3 or 5. Accordingly, in our study, acceptable detectability of lung lesions was achieved in all low-dose groups when ADMIRE 4 was applied.

This study has some limitations. First, in selected young adults with chronical diseases such as cystic fibrosis, treatment is still performed in our Department of Pediatrics and Adolescent Medicine. Consequently, a minor portion of subjects of our study groups was older than 18 years. However, no bias is expected on this issue, since none of the adult patients exceeded adolescents in height or BMI. Second, we included examinations with and without intravenous injection of contrast media in the study. Third, we cannot provide sensitivity of lung lesion detection since no internal reference standard was available. Instead, we evaluated diagnostic confidence and subjective image quality of both anatomical lung structures and detected suspicious lung lesions. Nevertheless, sensitivity regarding detection of pulmonary nodules is known to be high. Messerli et al. [24] detected lung nodules in adults with a sensitivity of 91.2% using a low-dose chest CT protocol comparable to our Sn64 protocol. In a phantom study performed by Grodic et al. [25], sensitivity of pulmonary nodule detection was 94% in low-dose groups (1/10th and 1/20th of standard dose, tin prefiltration, ADMIRE). Although results of sensitivity resulting from the studies mentioned before cannot be assigned to our collective, they at least tend to support the validity of our findings.

Further research is needed in this area, especially with regard to subtle pathologies such as very small pulmonary metastases or minor interstitial pathologies, which was not part of our study. In our hospital, the Sn32 protocol has mainly been established for follow-up studies of noncancer patients. In our opinion, it should not be used in patients with suspicion of subtle pathologies as mentioned above because of the potential limitations in image quality.

## 5. Conclusions

In DSCT examinations of the pediatric and adolescent lung, spectral shaping together with advanced iterative reconstruction enables substantial radiation dose reduction. Despite a certain reduction in overall diagnostic confidence and image quality of small anatomical structures, dose lowering to about 5% of a full-dose protocol still enables acceptable detectability of small lung lesions. The remaining radiation dose is about 10-fold higher compared to a single plane DR. If unlimited DSCT image quality is not mandatory, the latter protocol may serve as an alternative to DR with the option of more precise detectability of pathological changes.

## Figures and Tables

**Table 1:** Patient characteristics.

Dose Group	Sn96	Sn64	Sn32	Digital Radiographs (DR)	*p*-Value *
Number of patients	19	19	19	19	
Gender	12 male, 7 female	12 male, 7 female	13 male, 6 female	12 male, 7 female	
Age: mean ± SD, median (range)	12.6 ± 8.0 12.9 (1.3–28.3)	13.9 ± 3.8 14.1 (5.6–18.9)	12.7 ± 4.9 13.7 (4.8–21.5)	12.8 ± 5.4 13.0 (2.9–22.5)	ANOVA: *p* = 0.956
Weight: mean ± SD	39.5 ± 21.6	49.3 ± 15.8	46.5 ± 22.8	39.9 ± 16.2	ANOVA: *p* = 0.497
Body mass index: mean ± SD	18.7 ± 4.5	18.9 ± 3.0	19.1 ± 4.8	17.3 ± 2.7	ANOVA: *p* = 0.620

Sn96/Sn64/Sn32: dose groups with tin prefiltration and different reference tube current time products (96/64/32 ref. mAs). * No significant differences (*p* < 0.05) were found between the groups.

**Table 2:** Diagnostic confidence and anatomical structures.

Analysed Item	Reconstruction	Sn96	Sn64	Sn32	DR	*p*-Values
Diagnostic confidence	FBP	2.3 ± 0.6	1.8 ± 0.6	1.3 ± 0.3		Sn96 vs. Sn64: *p* = 0.115; Sn96 vs. Sn32: *p* < 0.001; Sn64 vs. Sn32: *p* = 0.003
	ADMIRE 2	2.7 ± 0.4	2.5 ± 0.4	1.9 ± 0.4		Sn96 vs. Sn64: *p* = 1; Sn96/Sn64 vs. Sn32: *p* < 0.001
	ADMIRE 3	3.3 ± 0.5	2.9 ± 0.4	2.3 ± 0.4		Sn96 vs. Sn64: *p* = 0.065; Sn96 vs. Sn32: *p* < 0.001; Sn64 vs. Sn32: *p* = 0.001
	ADMIRE 4	3.4 ± 0.5	3.2 ± 0.5	2.7 ± 0.6	2.4 ± 0.5	Sn96 vs. Sn64: *p* = 1; Sn96 vs. Sn32: *p* < 0.001; Sn64 vs. Sn32: *p* = 0.013
						**DR vs. Sn96/Sn64: *p* < 0.001; DR vs. Sn32: *p* = 0.505**
Medium-sized vessels	FBP	3.6 ± 0.7	3.3 ± 0.4	2.3 ± 0.5		Sn96 vs. Sn64: *p* = 0.867 Sn96/Sn64 vs. Sn32: *p* < 0.001
	ADMIRE 2	3.7 ± 0.6	3.6 ± 0.4	3.0 ± 0.6		Sn96 vs. Sn64: *p* = 1; Sn96 vs. Sn32: *p <* 0.001; Sn64 vs. Sn32: *p* = 0.005
	ADMIRE 3	3.8 ± 0.5	3.8 ± 0.4	3.1 ± 0.6		Sn96 vs. Sn64: *p* = 1 Sn96/Sn64 vs. Sn32: *p <* 0.001
	ADMIRE 4	3.8 ± 0.4	3.9 ± 0.2	3.4 ± 0.6	2.6 ± 0.6	Sn96 vs. Sn64: *p* = 0.872; Sn96 vs. Sn32: *p* = 0.168; **Sn64 vs. Sn32: *p* = 0.021**
						**DR vs. Sn96/Sn64: *p* < 0.001;** **DR vs. Sn32: *p* = 0.001**
Small vessels	FBP	2.4 ± 0.6	2.1 ± 0.4	1.3 ± 0.4		Sn96 vs. Sn64: *p* = 0.229 Sn96/Sn64 vs. Sn32: *p* < 0.001
	ADMIRE 2	3.1 ± 0.6	2.7 ± 0.5	2.0 ± 0.7		Sn96 vs. Sn64: *p* = 0.330; Sn96 vs. Sn32: *p* < 0.001; Sn64 vs. Sn32: *p* = 0.001
	ADMIRE 3	3.1 ± 0.6	3.0 ± 0.4	2.1 ± 0.4		Sn96 vs. Sn64: *p* = 1 Sn96/Sn64 vs. Sn32: *p* < 0.001
	ADMIRE 4	3.2 ± 0.6	3.1 ± 0.4	2.5 ± 0.5	1.9 ± 0.6	Sn96 vs. Sn64: *p* = 1; Sn96 vs. Sn32: *p* = 0.001; **Sn64 vs. Sn32: *p* = 0.002**
						**DR vs. Sn96/Sn64: *p* < 0.001;** **DR vs. Sn32: *p* = 0.004**
Lung parenchyma	FBP	2.1 ± 0.7	1.6 ± 0.5	1.2 ± 0.3		Sn96 vs. Sn64: *p* = 0.094; Sn96 vs. Sn32: *p* < 0.001; Sn64 vs. Sn32: *p* = 0.028
	ADMIRE 2	2.7 ± 0.5	2.6 ± 0.5	1.8 ± 0.4		Sn96 vs. Sn64: *p* = 1 Sn96/Sn64 vs. Sn32: *p* < 0.001
	ADMIRE 3	2.7 ± 0.4	2.5 ± 0.5	2.1 ± 0.4		Sn96 vs. Sn64: *p* = 0.881; Sn96 vs. Sn32: *p* = 0.003 Sn64 vs. Sn32: *p* = 0.097
	ADMIRE 4	2.9 ± 0.5	2.9 ± 0.4	2.7 ± 0.5	1.2 ± 0.3	Sn96 vs. Sn64: *p* = 1; Sn96 vs. Sn32: *p* = 0.375; Sn64 vs. Sn32: *p*= 0.807
						**DR vs. Sn96/Sn64/Sn32: *p* < 0.001**
Lung fissures	FBP	2.2 ± 0.6	2.2 ± 0.5	1.4 ± 0.6		Sn96 vs. Sn64: *p* = 1 Sn96/Sn64 vs. Sn32: *p* < 0.001
	ADMIRE 2	2.8 ± 0.6	2.6 ± 0.7	1.8 ± 0.7		Sn96 vs. Sn64: *p* = 1; Sn96 vs. Sn32: *p <* 0.001; Sn64 vs. Sn32: *p* = 0.001
	ADMIRE 3	2.8 ± 0.7	2.7 ± 0.6	2.0 ± 0.6		Sn96 vs. Sn64: *p* = 1; Sn96 vs. Sn32: *p* = 0.001; Sn64 vs. Sn32: *p* = 0.002
	ADMIRE 4	2.8 ± 0.7	2.9 ± 0.6	2.3 ± 0.5	1.4 ± 0.4	Sn96 vs. Sn64: *p* = 1; Sn96 vs. Sn32: *p* = 0.014; **Sn64 vs. Sn32: *p* = 0.001**
						**DR vs. Sn96/Sn64/Sn32: *p* < 0.001**
Tertiary bronchi	FBP	3.2 ± 0.6	2.8 ± 0.6	2.2 ± 0.5		Sn96 vs. Sn64: *p* = 0.314; Sn96 vs. Sn32: *p* < 0.001; Sn64 vs. Sn32: *p* = 0.002
	ADMIRE 2	3.6 ± 0.5	3.4 ± 0.5	3.2 ± 0.6		Sn96 vs. Sn64: *p* = 1; Sn96 vs. Sn32: *p* = 0.034; Sn64 vs. Sn32: *p* = 0.511
	ADMIRE 3	3.8 ± 0.4	3.8 ± 0.4	3.3 ± 0.6		Sn96 vs. Sn64: *p* = 1; Sn96 vs. Sn32: *p* = 0.02; Sn64 vs. Sn32: *p* = 0.004
	ADMIRE 4	3.8 ± 0.4	3.7 ± 0.4	3.5 ± 0.5	1.6 ± 0.4	Sn96 vs. Sn64: *p* = 1; Sn96 vs. Sn32: *p* = 0.182; Sn64 vs. Sn32: *p* = 0.611
						**DR vs. Sn96/Sn64/Sn32: *p* < 0.001**

Ratings on a 4-point Likert scale (1 = unacceptable, 2 = acceptable under limited conditions, 3 = probably acceptable, 4 = fully acceptable) of posterior–anterior digital radiographs (DR) and dual-source computed tomography (DSCT) groups (Sn96/Sn64/Sn32: tin prefiltration and different ref. mAs) with different reconstruction algorithms (filtered back-projection (FBP)/ADMIRE 2/3/4). Values are given as mean ± standard deviation. *p* < 0.05 was considered to be statistically significant.

**Table 3:** Detectability, contrast and contour sharpness of lesions.

	Sn96_ADM4_	Sn64_ADM4_	Sn32_ADM4_	DR	*p*-Value *
Number of lesions	74	68	67	68	
Lesions per patient	3.7	3.6	3.6	3.6	
Size (mm)	5.1 ± 4.3	5.9 ± 5.8	7.0 ± 5.5	5.9 ± 1.8	**DR vs. Sn96 vs. Sn64 vs. Sn32: ANOVA *p* = 0.121**
Detectability	3.4 ± 0.6	3.3 ± 0.7	3.0 ± 0.6	1.9 ± 0.8	**DR vs. Sn96/Sn64/Sn32: *p* < 0.001** Sn96 vs. Sn64: *p* = 0.779 Sn64 vs. Sn32: *p* = 0.343 Sn96 vs. Sn32: *p* = 0.009
Contrast	2.9 ± 0.7	2.9 ± 0.6	2.7 ± 0.7	2.0 ± 0.6	**DR vs. Sn96/Sn64/Sn32: *p* < 0.001** Sn96 vs. Sn64: *p* = 0.969 Sn64 vs. Sn32: *p* = 0.873 Sn96 vs. Sn32: *p* = 0.378
Contour sharpness	2.9 ± 0.7	2.8 ± 0.7	2.4 ± 0.7	1.9 ± 0.7	**DR vs. Sn96/Sn64/Sn32: *p* < 0.001** Sn96 vs. Sn64: *p* = 0.814 Sn64 vs. Sn32: *p* = 0.006 Sn96 vs. Sn32: *p* < 0.001

Rated on a 4-point Likert scale (1 = unacceptable, 2 = acceptable under limited conditions, 3 = probably acceptable, 4 = fully acceptable). Values are given for the DR group (posterior–anterior) and Sn96_ADM4_/Sn64_ADM4_/Sn32_ADM4_ groups (tin prefiltration, 96/64/32 reference mAs, reconstruction with ADMIRE 4). * In case of significant differences in one-way ANOVA (*p* < 0.05), post hoc pairwise comparisons are displayed.

**Table 4:** Radiation dose estimations.

	Sn96	Sn64	Sn32	DR	*p*-Value *
CTDI_Vol_ (mGy)	0.34 ± 0.18	0.25 ± 0.09	0.13 ± 0.08		Sn96 vs. Sn64: *p* = 0.13; Sn96 vs. Sn32: *p* < 0.001; Sn64 vs. Sn32: *p* = 0.011
DLP (mGy∗cm)	11.5 ± 7.5	9.0 ± 3.7	4.0 ± 2.6		Sn96 vs. Sn64: *p* = 0.371; Sn96 vs. Sn32: *p* < 0.001; Sn64 vs. Sn32: *p* = 0.010
DAP (cGy∗cm^2^)				1.57 ± 0.75	
D_E_ (mGy)				0.015 ± 0.016	
ED (mSv)	0.219 ± 0.096	0.149 ± 0.054	0.065 ± 0.035	0.007 ± 0.003	Sn96/Sn64/Sn32 vs. DR: *p* < 0.001 Sn96 vs. Sn64: *p* = 0.094
ED of CT groups in multiples of DR	**31**	**21**	**9**	**1**	

Sn96/Sn64/Sn32 groups with tin prefiltration and different reference tube current time products (96/64/32 ref. mAs). Posterior–anterior digital radiographs (DR). Values of volumetric CT dose index (CTDI_Vol_), dose length product (DLP), dose area product (DAP), entrance dose (D_E_) and effective dose (ED) are given as mean ± standard deviation. * Significant differences in ED were found between all Sn groups and DR.

## Data Availability

All data generated and analyzed during this study are included in this published article. Raw data supporting the findings of this study are available from the corresponding author on request.
